# Radiation induced malignant histiocytoma of the contralateral breast following treatment of breast cancer: a case report and review of the literature

**DOI:** 10.1186/1757-1626-1-313

**Published:** 2008-11-17

**Authors:** Saptarshi Biswas, Faruq Badiuddin

**Affiliations:** 1 Department of General Surgery, Stanford University Medical Center. 300 Pasteur Drive, Palo Alto, CA- 94305, USA; 2 Emirates Society of Laparo-Endoscopic Surgery, UAE University, Dubai, UAE

## Abstract

Radiation therapy (XRT) is an important modality for treatment of breast cancer. Its use has occasionally resulted in the development of secondary malignancies.

We describe this interesting case of a 46-year-old woman who developed malignant fibrous histiocytoma in the contralateral breast 6 years after a lumpectomy followed by radiation therapy for infiltrating ductal carcinoma. The role of XRT in the treatment of breast carcinoma and development of Radiation induced Sarcoma (RIS) is examined.

RIS has a current incidence of 0.03% to 0.2% in patients undergoing XRT for breast carcinoma. The role of XRT in the development of RIS has been clearly demonstrated. Clinical presentations vary, and diagnosis is commonly delayed. Treatment consists of wide surgical excision. Development of RIS has an average latency of over 10 years and correlates with the dose and technique of XRT. Breast conserving surgery followed by irradiation is becoming increasingly popular leading to an increasing number of sarcomas. Because of post-irradiation changes, detection of a new lesion is difficult, resulting in delayed diagnosis and poor prognosis in these patients. However, the benefit of XRT far outweighs the risk of RIS and should not affect the decision to treat these patients with this modality.

## Background

Radiation Induced Sarcomas were described following treatment for tuberculous arthritis and in workers painting radium watch dials. Cahan et al [1] first described (1948) the RIS diagnosis criteria which included a prior history of radiation, latency period of 5 years/more, the development of sarcoma within a previously irradiated field, and a histological confirmation of sarcoma. These criteria was later modified to include the tissues adjacent to the radiated field and a shorter latency period of 3–4 years still continue to define RIS. The incidence of sarcomas in breast cancer patients following mastectomy and chest wall irradiation is reported to be 0.2% at 10 years [2].

RIS was first described in the setting of radical mastectomies, and was later in patients undergoing lumpectomy and radiation therapy. As breast conserving therapy becomes more prevalent, it will be important to monitor the frequency of this complication.

We present a rare case of RIS in the contralateral breast out of the field of radiation, after radiotherapy had been administered 6 years earlier to the other side.

## Case report

**Figure 1:**
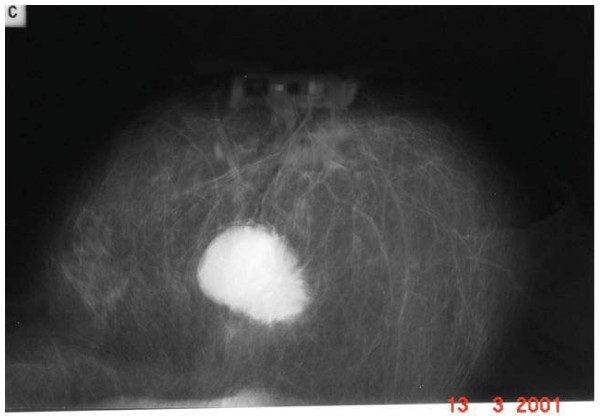
Mammographic picture of breast lesion.

**Figure 2:**
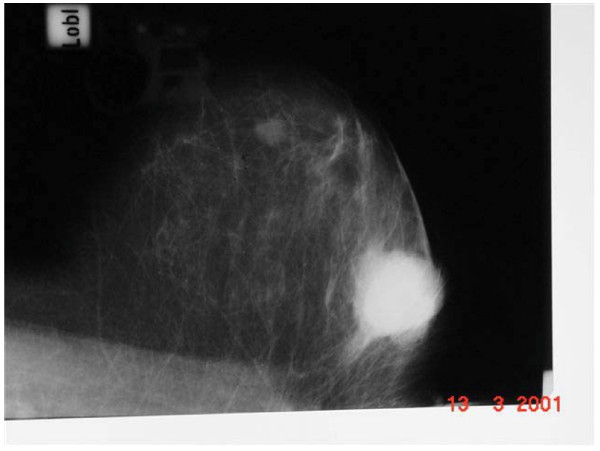
Mammographic picture of breast lesion.

**Figure 3:**
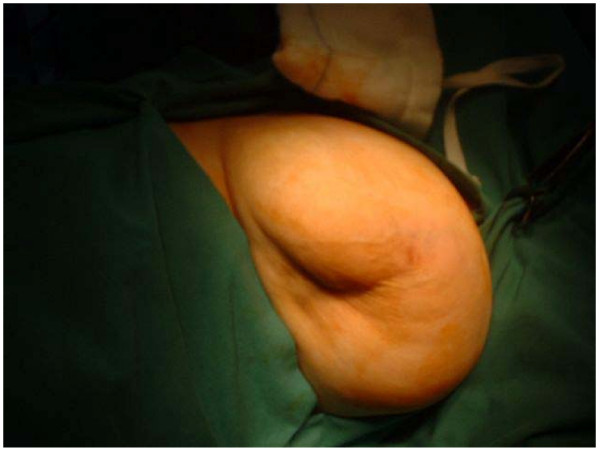
Gross appearance of Breast lesion.

A 46 year old Caucasian woman was seen in the breast clinic in Tralee General Hospital, Tralee, County Kerry, Republic of Ireland (March, 95) with a 3 × 2 cms right breast lump. Bilateral mammography followed by open biopsy confirmed an adenocarcinoma in the upper outer quadrant of the right breast. Left breast was normal. The patient underwent simple mastectomy followed by radiotherapy to right chest wall, axilla and supra-clavicular fossa and chemotherapy with Cyclophosphamide, Methotrexate and 5-FU. In February 2001 the same patient presented with a lump in the contralateral breast. The lump, present for 6 months, was non-tender and slow growing. Mammography figures as shown (Figures 1 and 2) Overlying skin tethering was present (Figure 3). No nipple discharge or axillary lymphadenopathy was noted.

Cytology was graded as C1. Trucut specimen histology showed cellular connective tissue with fibroblasts and enlarged, hyperchromatic nuclei. Amid those desmoplasia were single histiocytes with granular foamy nuclei. There was also cellular dense collagen involving adipose tissue. Fibroblast proliferation accompanied by collagen necrosis was extensive and was reported as post radiation change.

The lump was excised. Specimen revealed a 3.2 cm central tumor with a cyst 2 cm in dimension within. The tumor arose in the soft tissue underlying the skin. A central cystic cavity surrounded by extensive tumor necrosis was noted along with a vascular tumor with fibrocellular nodules surrounded by a network of collagen fibres. Some of the nodules were hypercellular containing frequent mitoses. There was an irregular infiltrating edge to the tumor into the adipose tissue. The tumour was classified as a malignant fibrous histiocytoma arising from the breast stroma/subcutaneous tissue.

This was based on the finding of predominant storiform pattern, and presence of epithelioid along with tumor giant cells. The tumor had 2 parts, majority of low grade differentiation. 1/3 rd was of high grade with >3 mitoses/high power field. The patient was underwent left mastectomy and axillary clearance. Histology confirmed tumour clearance and no evidence of axillary node metastasis. Body scan revealed no distant metastases.

## Discussion

Post radiation changes following breast carcinoma treatment include arm edema, brachial plexopathy, decreased arm mobility, soft tissue necrosis, rib fractures, radiation pneumonitis, radiation related heart disease and carcinogenesis [3].

Primary malignant fibrous histiocytoma of the female breast are rare. Clinically, radiation associated sarcomas present as cutaneous lumps within the previously irradiated area of the chest wall (parasternal area, supraclavicular fossa, shoulder girdle and conserved breast). Mammography is typically negative [3-5] and the mean latency period approximately 11 years (4 to 44 years).

Patients who developed RIS were young [6] when diagnosed with breast cancer (range 26 – 54 ; median 43 years) compared to patients with lymphangiosarcoma (range 39 to 69 years, median 51 years) and the general breast cancer population. The median age in a separate study carried out at Institut Gustave Roussy (France) in comparison were 65.8 years (49 – 83 years) and the mean latent period was 9.5 years (4–24 years). The cumulative incidence of sarcoma following irradiation of breast cancer was 0.2% (range 0.09 to 0.47) at 10 years. In a separate retrospective analysis of RIS following XRT for breast cancer at Henri Mondor Hospital, France (1983 – 1997) [7] RIS appeared with a latency period of 5 to 18 years (mean 10.3 years) and the mean age being 57.6 years (range 39 to 88 years).

Few studies have addressed the risk of contralateral breast cancer after post operative RT. In a large cohort study of 41,109 patients diagnosed with breast cancer (1935 – 1982). RT was associated with a small but marginally significant elevation in the risk of the contralateral breast cancer [8] Patients aged 45 years or younger the risk of contralateral breast cancer within 15 years was found to be 11% without RT and increased to about 12–13% with the addition of RT. The quantitative risk of Radiation induced sarcoma following breast conserving radiotherapy was no greater than that following mastectomy. Patients receiving both XRT and chemotherapy was noted to be at highest risk for secondary malignancies, including sarcomas and leukaemias.

Radiation induced neoplastic transformation is thought to be related to irreversible DNA damage [9]. Several years following radiotherapy, dominant gene mutations and gene deletions accumulate in the genome, making carcinogenesis a multistage process. Cells in G2 and M phases of the cell cycle [10] are radiosensitive in terms of both killing and induction of neoplastic transformation compared with mid G1 phase. The exact molecular mechanisms of tumor promotion by ionizing radiation are unknown. Proto-oncogene c-jun expression [11] and inactivation of tumor suppressor genes p53 and Rb are commonly discussed theories. The retinoblastoma locus may be important in the pathogenesis of soft tissue sarcomas. Retinoblastoma gene alterations have been detected in de novo leiomyosarcomas as well as RIS.

It is difficult to analyze the exact relationship between the total irradiation dose and RIS. Minimum total doses of 10 Gy in conventional doses per fraction appear necessary to result in RIS, most cases of RIS occur in association with total radiation doses in the range of 40 – 50 Gy [12]. The risk of carcinogenesis increases linearly with doses up to 10 Gy. Pierce et al [13] suggested a relationship between radiation technique, potential overlapping of fields, and the development of secondary tumors.

Current radiation therapy regimens consist of delivery of 5000 cGy to the whole breast at a dosage of 200 c Gy/day, additional 1000–1500 cGy to the tumor bed. Postmastectomy radiation is mainly reserved for patients with T3 or T4 primary tumours or multiple positive lymph nodes.

Radiation associated sarcomas are usually high grade tumors [6] reflected by the advanced stage at the time of diagnosis. Pathological diagnosis is often delayed due to lack of symptoms, distortion of histological architecture and long latency period after diagnosis of the original tumor.

## Treatment

The treatment of choice for sarcomas of any histological type, de-novo or post radiation exposure is a wide margin resection, which can be challenging in chest wall sarcomas because of proximity of vital structures. Centrally located sarcomas often recur due to resection inadequacy. Advanced soft tissue sarcomas need multimodality therapy, surgical resection with chemotherapy and at times radiotherapy are necessary. Okuno et al [14] has published an extensive treatment review of advanced soft tissue sarcomas with systemic therapy. Survival time varied between 10–48 months [15] This poor survival was due to delay in diagnosis, aggressive local nature of tumors and truncal location, making radical extirpative surgery technically difficult. Experience with adjuvant chemotherapy in RIS is limited and disappointing. Some investigators believe that chemotherapy are of limited effectiveness in RIS due to fibrotic tissue changes in the previously irradiated field, thus preventing the chemotherapy from reaching adequate concentrations in the target organ.

## Conclusion

Although the role of radiation therapy in inducing the development of sarcomas is evident, this risk is not increasing with time, and the benefit offered by irradiation in the treatment of breast cancer far outweighs the risk of secondary malignancies and should not affect the decision to treat a breast cancer patient with adjuvant XRT. The long latency period, difficulty in detecting the tumor clinically and histologically in previously irradiated tissue (e.g., due to fibrosis) and inadequate biopsies makes the detection and diagnosis of this condition a challenge. Therefore, a high level of suspicion, careful patient evaluation and adequate biopsy tissue for pathologic diagnosis are mandatory.

## Competing interests

The authors declare that they have no competing interests.

## Authors' contributions

FB was the primary surgeon and SB was the assisting surgeon and the primary author.
